# Incidence and Management of Cerebrospinal Fluid Leakage due to Late Presentation of Dural Tears After Lumbar Surgery

**DOI:** 10.1111/os.70348

**Published:** 2026-06-02

**Authors:** Xiaoxiong Yang, Dazhao Tie, Rui Chen, Xin Chen, Yanbin Zhao, Hua Zhou, Yan Li, Lei Dang, Chuiguo Sun, Feifei Zhou

**Affiliations:** ^1^ Orthopedic Department Peking University Third Hospital Beijing China; ^2^ Engineering Research Center of Bone and Joint Precision Medicine Peking University Beijing China; ^3^ Beijing Key Laboratory of Spinal Disease Research Peking University Beijing China

**Keywords:** cerebrospinal fluid leakage, late presentation of dural tears, lumbar spine, postoperative complications

## Abstract

**Objective:**

To investigate the incidence and treatment of cerebrospinal fluid leakage due to late presentation of dural tears (LPDT) after lumbar surgery and provide new ideas and treatment options for clinical management. This study addresses a significant knowledge gap in the specific context of delayed CSF leaks, where diagnosis and management remain particularly challenging despite existing literature on CSF leaks in general.

**Methods:**

Patients who underwent posterior lumbar surgery from January 2021 to December 2024 were selected. General patient information, cerebrospinal fluid leakage (CSF) characteristics, follow‐up results, and re‐examination data were analyzed using chi‐squared tests, Fisher's exact tests, *t*‐tests, and ANOVAto identify cases of LPDT, their treatment methods, complications, and clinical outcomes.

**Results:**

A total of 2359 patients who underwent lumbar surgery were included in the study. CSF leakage occurred in 43 patients (1.82%). Dural tears (DT) leading to CSF leakage intraoperatively or within 5 days postoperatively were observed in 36 patients (1.53%), with 23 (0.98%) identified intraoperatively. Seven patients (0.30%) experienced CSF leakage due to LPDT: two of them underwent dural repair surgery 3 weeks and 4 months postoperatively, respectively; five patients received conservative treatment involving wound pressure bandage and bed rest. Clinical outcomes were favorable (good or excellent) in five patients (71.4%) and poor in two patients (28.6%) with LPDT, whereas in contrast, no poor outcomes were observed in non‐LPDT patients.

**Conclusion:**

This study found a 0.30% incidence of CSF leakage due to LPDT following lumbar surgery, with these patients demonstrating worse outcomes compared to those with nondelayed CSF leaks. CSF leakage secondary to unrecognized LPDT is an uncommon but clinically significant complication of spine surgery, necessitating heightened awareness and appropriate management.

## Graphical abstract

This article reveals a 0.3% incidence of LPDT and highlights the importance of increased clinical awareness. Furthermore, appropriate management to optimize patient recovery and minimize long‐term complications.

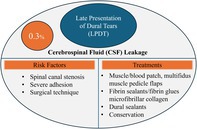

## 1 Introduction

Dural tear (DT) with resultant cerebrospinal fluid (CSF) leakage is a recognized complication of spinal surgery, with reported incidences ranging from 1% to 17% [1, 2], most DTs can be identified and addressed intraoperatively. However, the literature on CSF leakage due to LPDT remains limited [3, 4]. LPDT following lumbar spine surgery is a complex complication that can arising days to weeks after the procedure [5, 6]. Characterized by symptoms such as headache, nausea, and vomiting, LPDT can significantly impact patient quality of life, potentially delaying recovery, increasing financial burden, and escalating healthcare costs [7, 8]. While wound healing, surgical technique, and postoperative management have been implicated, a comprehensive understanding of the underlying mechanisms and influencing factors remains incomplete [9, 10]. Current treatment strategies, including conservative management and surgical repair like applying muscle or blood patch grafts, microfibrillar collagen [4, 11], are limited by delayed diagnosis and inconsistent efficacy.

While previous studies have examined postoperative complications in lumbar surgery, the specific epidemiology and optimal management strategies for LPDT remain incompletely characterized. Most existing large‐scale studies have focused on intraoperative or early postoperative CSF leaks, leaving delayed presentations less understood. Currently, few studies compare the clinical outcomes of LPDT directly with early‐presenting CSF leaks. Additionally, the mechanisms, symptom timelines, and criteria for choosing between surgical and conservative treatments in delayed cases remain unclear. Therefore, this study uses a large cohort to compare LPDT with non‐delayed CSF leaks, aiming to better describe their clinical course and provide practical data for managing these late‐presenting cases.

## 2 Materials and Methods

### 2.1 Patient Population and Selection Criteria

This study included patients who underwent posterior lumbar surgery at our hospital between January 2021 and December 2024. Inclusion criteria were patients with degenerative lumbar spine disease without cauda equina syndrome. Exclusion criteria were: (i) lumbar scoliosis or kyphotic deformities; (ii) ankylosing spondylitis and rheumatoid arthritis; (iii) lumbar spine infections and systemic chronic infections; (iv) previous spinal surgery at the same level within 6 months. A total of 2359 patients met the inclusion and exclusion criteria. The minimum postoperative follow‐up period was 3 months to confirm wound healing and the presence of delayed CSF leakage, with an average follow‐up of 6 months.

### 2.2 Diagnostic Criteria for LPDT

LPDT was defined based on the following criteria from previous literature [12]: (i) Symptoms of low CSF pressure, such as orthostatic hypotension, photophobia, and nausea, appearing from the 5th postoperative day; (ii) Clear drainage fluid from the wound; (iii) MRI confirming CSF leakage; (iv) presence of ±β2‐microglobulin ferritin, in conjunction with the first three diagnostic criteria, may indicate LPDT. The fourth criterion was considered supportive but not essential for diagnosis when the other three criteria were met.

While MRI confirmation was part of our diagnostic criteria, in clinical practice, when patients presented with characteristic symptoms (orthostatic headache, clear wound drainage) and responded to conservative management, the diagnosis of LPDT was made even without MRI confirmation in some cases. Therefore, therapeutic efficacy demonstrated through symptom resolution following timely intervention provides sufficient diagnostic validation for LPDT, obviating the necessity for MRI confirmation.

### 2.3 Data Collection and Statistical Analysis

Patient characteristics and clinical information were extracted from the medical records and analyzed in MS Excel (Microsoft, Redmond, Washington, USA). Statistical analyzes were performed using the SPSS software (SPSS 24.0 for Windows; IBM, Armonk, New York, USA). Categorical variables are presented as percentages and counts; values are presented as mean ± standard deviation or number (%), and the difference between rates was tested by chi‐squared test or Fisher exact tests, as appropriate. Quantitative variables were tested using one‐way ANOVA or the Kruskal‐Wallis test if data were not normally distributed. The “Statistic Value” in tables represents the *χ* ^2^ (chi‐squared) value for categorical data comparisons; statistical significance was set at *p* < 0.05.

## 3 Results

### 3.1 Patient Demographics and Surgical Characteristics

The study population included patients with LCS (lumbar canal stenosis, LCS) (*n* = 1686), LDH (Lumbar Disc Herniation, LDH) (*n* = 480), and DLS (Degenerative Lumbar Spondylolisthesis, DLS) (*n* = 193). Surgical procedures included total laminectomy (*n* = 1998), fenestration decompression (*n* = 293), and revision surgery (*n* = 68). Surgical levels were classified into ≤ 2 levels (*n* = 695), > 2 and ≤ 4 levels (*n* = 1073), and > 4 levels (*n* = 593). Data on intraoperative blood loss and surgery duration were collected.

Statistical analysis showed significant differences in surgical methods, surgical level, surgery duration, and intraoperative blood loss between patients with and without cerebrospinal fluid leakage (*p* < 0.05) (Table 1).

**TABLE 1:** General characteristics and surgical outcomes of patients with and without cerebrospinal fluid leaks.

General information (*N* = 2359)		Cerebrospinal fluid leak group (*N* = 43, 1.82%)	Noncerebrospinal fluid leak group (*N* = 2316, 98.18%)	*p*	*t*
Gender (Male/Female)		20/23	1184/1132	*p* = 0.37	0.55
Age (years)		53.73 ± 9.77	54.83 ± 8.43	*p* = 0.438	0.845
BMI (kg/m^2^)		24.39 ± 4.68	25.06 ± 5.71	*p* = 0.445	0.765
Preoperative diagnosis	LCS	29	1657	*p* = 0.67	0.18
	LDH	10	470		
	DLS	4	189		
Surgical method	Total laminectomy	34	1964	*p* = 0.002*	12.13
	Fenestration	4	289		
	Revision	5	63		
Segments	≤ 2	23	672	*p* = 0.001*	13.58
	2 < L ≤ 4	16	1057		
	> 4	4	587		
Surgical duration (min)		206.18 ± 35.74	276.57 ± 58.26	*p* = 0.001*	7.894
Intraoperative blood loss (mL)		183.39 ± 32.38	152.06 ± 21.45	*p* = 0.001*	9.384
Drainage tube retention time (h)		134.65 ± 18.73	81.49 ± 16.35	*p* = 0.001*	21.067
Hospital stay time (h)		197.52 ± 25.61	103.45 ± 30.24	*p* = 0.001*	20.263

*Note:* Percentages for CSF leak group are calculated based on *N* = 43, for nonleak group based on *N* = 2316. *means *p* < 0.05, statistically significant difference.

### 3.2 Incidence and Prognostic Features of LPDT

Reviews of 43 CSF leak patients revealed a 1.53% incidence of dural tears (DTs) with CSF leaks occurring intraoperatively or within 5 days postsurgery. Intraoperative DTs were observed in 23 cases (0.98%), with nine undergoing dural repair. LPDToccurred in seven cases (0.30%). Among LPDT patients, five demonstrated good or excellent recovery, while two experienced poor outcomes, in contrast to nondelayed CSF leak patients who did not exhibit poor recovery outcomes (Tables 2 and 3).

**TABLE 2:** Management and interventions for cerebrospinal fluid leak.

Cerebrospinal fluid leak features		Yes(% of total cohort, *N* = 2359)	Yes (% of CSF leak group, *N* = 43)	No(% of total cohort, *N* = 2359)	Statistic value (*χ* ^2^)	*p*
Delayed cerebrospinal fluid leak		7 (0.30%)	7 (16.3%)	36 (1.53%)	6.153	0.013
Intraoperative discovery		23 (0.98%)	23 (53.5%)	11 (0.47%)	0.242	0.622
Intraoperative management measures	Simple dura mater closure	9 (0.38%)	9 (20.9%)	23 (9.75%)	12.914	0.000
	Dura mater closure + free muscle flap	3 (0.13%)	3 (7.0%)	—		
	Dura mater closure + patch	2 (0.01%)	2 (4.7%)	—		
	Lumbar cistern drainage	2 (0.08%)	2 (4.7%)	41 (1.74%)	5.349	0.021
Wound Infection		5 (0.21%)	5 (11.6%)	40 (1.70%)	4.092	0.043

*Note:* The wound infection total is 45 because it includes 2 patients from the non‐CSF leak group who developed wound infections unrelated to CSF leakage.

**TABLE 3:** Prognosis differences between the LPDT group and No‐LPDTgroup.

Prognostic outcome	LPDT(*N* = 7)	LPDT (% of LPDT group)	No‐LPDT(*N* = 36)	No‐LPDT (% of No‐LPDT group)	Statistic value (*N* = 36)	*p*
Excellent	1	14.3%	24	66.7%	14.849	0.000
Good	3	42.9%	9	25.0%	1.591	0.207
Fair	1	14.3%	3	8.3%	N/A	N/A
Poor	2	28.6%	0	0.0%	N/A	N/A

### 3.3 Clarification of Patient Groups

Among the 36 non‐LPDT CSF leak patients, 23 had intraoperatively recognized dural tears, while 13 experienced CSF leaks within 5 days postoperatively due to either unrecognized intraoperative tears that manifested early or possible repair failures. The LPDT group (*n* = 7) specifically represents cases where CSF leakage symptoms first appeared after postoperative day 5.

**FIGURE 1:**
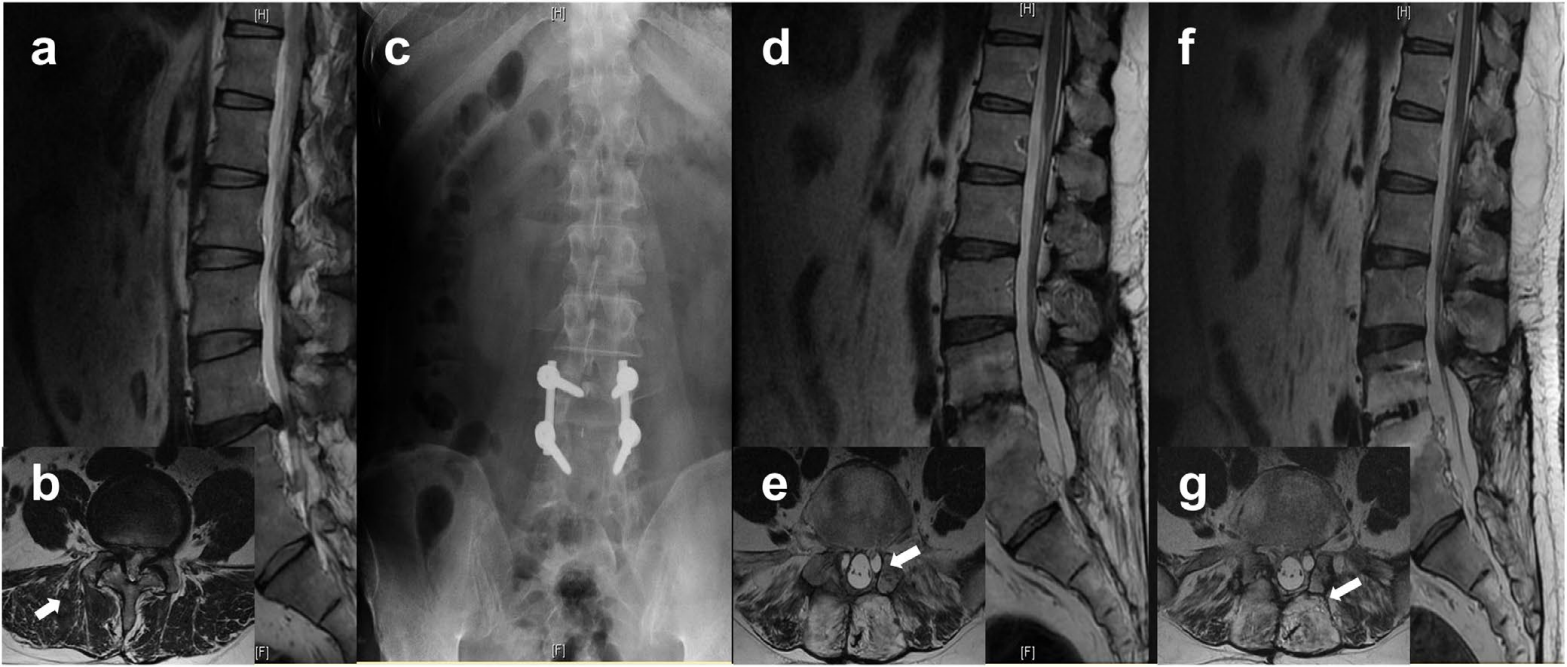
(a, b) Depicts the preoperative MRI manifestations. (c) Presents the postoperative lumbar X—ray. (d, e) Illustrates the manifestations of intraspinal pseudocyst (denoted by white arrows) 3 months after the surgery. Meanwhile, (f, g) demonstrated the intraspinal pseudocyst and the signal changes of paravertebral muscles 3 years after the surgery.

Detailed patient data for LPDT cases, including demographics, time of LPDT occurrence, surgical approach, symptoms, treatment modalities, follow‐up duration, and clinical outcomes, are presented in Table 4. MRI findings indicated the presence of CSF and a pseudomeningocele in the epidural space at the surgical site. Degenerative changes were also observed in the surrounding muscles and soft tissues (Figure 1).

**TABLE 4:** Detailed patient data for LPDT cases.

Gender/age + diagnosis	Surgical procedure	Intraoperative condition	Time to LPDT	Clinical presentation	MRI findings	Treatment	Follow‐up time	Outcome
Female/46 LDH	L4‐5 PLIF	Severe nerve adhesion	6 days	Headache, Nausea, Vomiting, Photophobia	Not examined	Wound pressure bandage, delay removal of drainage tube	3 months	Excellent
Male/47 LCS + DLS	L5‐S1 TLIF	Severe spinal canal stenosis	2 weeks	Headache, Nausea	Dural cyst with CSF leakage	Second surgery: Dura mater repair suturing and artificial dural patch	12 months	Poor
Female/50 LCS + LDH	L4‐5 PLIF	Large disk herniation with severe nerve root compression and adhesion	1 week	Headache, Nausea, Vomiting	Not examined	Compression bandage	6 months	Good
Female/66 LCS + DLS	L5‐S1 TLIF	Severe nerve compression and adhesion	2 weeks	Nausea	Not examined		6 months	Good
Male/58 LCS, Post‐op	L3‐4 Revision TLIF	Severe spinal cord and never root heavy adhesion	8 days	Headache	Not examined		9 months	Good
Male/47 LCS + LDH	L4‐5 PLIF	Large disk herniation and adhesion	2 months	Headache, Nausea, Photophobia	Epidural hematoma with CSF leak, pseudo‐dural cyst	Second surgery: Dural repair +muscle flap transfer + dural sealant	2 years	Poor
Male/63 LCS	L3‐4PLIF	Severe spinal canal stenosis	3 months	Headache, Photophobia	Paravertebral cerebrospinal fluid leakage	Bed rest	12 months	Fair

Abbreviations: DLS, degenerative lumbar spondylolisthesis; DT, dural tears; LCS, lumbar canal stenosis; LDH, lumbar intervertebral disk herniation; LPDT, late presentation of dural tears; PLIF, posterior lumbar interbody fusion; TLIF, transforaminal lumbar interbody fusion.

### 3.4 LPDT Treatment and Outcomes

Most CSF patients were managed conservatively. Intraoperative CSF leaks were addressed with simple dural suture (nine cases), dural suture with dural patch (three cases), or dural suture with free muscle flap (two cases). Postoperative CSF leaks occurring immediately or within 5 days were managed with local pressure dressings, normal pressure drainage, delayed extubation, and suture placement (Table 2). Two non‐LPDT patients underwent lumbar cistern drainage. Wound healing complications (three cases) were resolved with anti‐inflammatory wound care over 4 weeks, with no central nervous system infections observed. LPDT‐related CSF leak patients followed for 6–12 months experienced wound infections (two cases), managed with symptomatic wound care. Two patients with severe nausea/vomiting and orthostatic hypotension underwent local debridement and dural repair at 3 weeks and 4 months postoperatively, revealing bony spurs from the distal lamina edge or superior articular process. Postoperative management included regular dressing changes and pressure dressings, leading to improved wound healing and discharge. Postoperatively, one non‐LPDT patient experienced a myocardial infarction on the first day and was subsequently transferred to the CCU for further management. A patient with diffuse idiopathic skeletal hyperostosis (DISH) and concurrent thoracic spinal stenosis experienced bilateral lower extremity neurological decline following lumbar spine surgery. Subsequently, thoracic decompression surgery was performed to address the stenotic pathology.

## 4 Discussion

### 4.1 Incidence of Delayed Cerebrospinal Fluid Leakage

Delayed CSF leakage is an uncommon but noteworthy complication in lumbar spine surgery. The incidence of CSF leakage after initial lumbar surgery ranges from 5.5% to 9.0%, while in revision surgeries, it increases from 13.2% to 21.0% [13, 14]. Hassanzadeh H reported a 14% incidence of clinically significant and concluded that intraoperative recognition and repairDTare essential [15]. Khazim et al. [16] reported a 0.83% incidence of LPDT following lumbar spine surgery. Delayed CSF leakage has also been reported in a small number of studies, A small subset of patients may experience immediate postoperative postural headache and photophobia, potentially indicating CSF leakage in an upright position [17]. Delayed identification of DT can occur, with CSF leak symptoms manifesting days, weeks, or even months postsurgery. Furthermore, some cases report the development of pseudo‐meningoceles presenting as localized lower back pain, radiculopathy, or postural headache weeks or months after the procedure [18]. Pneumocephalus and pneumorachishave been reported after LPDT [5], therefore spontaneous spinal CSF leak should be taken into consideration in patients with recalcitrant orthostatic headaches after spine surgery, even if symptoms of the leak occur within hours of the spinal procedure [6]. following surgical intervention, lumbar drainage, or conservative management, all patients experienced favorable outcomes.

Our research indicates a 0.3% incidence of LPDT. However, this figure likely underestimates the true prevalence due to the asymptomatic nature of many cases of delayed CSF leaks. Clinicians should maintain a high index of suspicion, especially in patients presenting with positional headaches, hypotension, photophobia, nausea, or clear/serosanguinous wound drainage after the fifth postoperative day.

### 4.2 Risk Factors

LPDTs are defined as intraoperatively unrecognized DTsin patients who initially present without typical postdural puncture headache, photophobia, or dizziness [16]. LPDTs manifest symptoms developing shortly after the procedure, potentially due to the propagation of an initially small or weak dural defect into a larger tear with subsequent CSF leakage during severe headache episodes, as suggested by research [10].

Several risk factors contribute to the occurrence of LPDT, including severe spinal canal stenosis, significant nerve adhesion, and extensive surgical manipulation [19]. These factors can increase the likelihood of dural tears going unnoticed during surgery, leading to LPDT [20]. One hypothesis suggests that severe spinal stenosis or significant adhesion between the dura and surrounding tissues can predamage the dura, making it thin and susceptible to tearing during decompression [21]. Another possibility involves an initial CSF leak concealed beneath the fascia, maintained in a homeostatic state where CSF production (0.3–0.6 mL/min) is balanced with a slow leakage, keeping the pressure around 100 mm H_2_O [22]. Sharp bone fragments or spurs at the decompression edge, coupled with increased CSF pressure from physical activity, can cause dural expansion against the lamina or bony resection margin, potentially leading to dural tears and CSF leaks [15]. To prevent these, routine inspection of the dura and vertebral canal edges for bony spurs before wound closure is crucial, as advocated by Khazim et al. [16]. Furthermore, evidence suggests that early postextubation or Valsalva‐inducing activities, such as sneezing or straining, constipation, and coughing which increased abdominal pressure can also elevate internal pressure, therefore raise the risk of LPDT.

Age‐related spinal degeneration, including ossification and ligamentum flavum hypertrophy, exacerbates spinal stenosis and increases the risk of dura mater injury during laminectomy using Kerrison rongeurs [23]. However, research showed that ultrasonic bone scalpel helps to reduce the incidence of iatrogenic durotomies [24]. A large multicenter US studyon LPDTidentified that decompression alone, and surgical duration exceeding 250 min as significant risk factors for LPDT [19]. In our clinical practice, meticulous exploration around decompression sites for sharp bone spurs impinging on the dura is routine before wound closure, particularly in cases of severe spinal stenosis or revision surgery; however, LPDT still occurs inevitably. For that reason, we believe that LPDT is not caused by a single factor but is associated with a combination of patient‐related factors, which include severe spinal canal stenosis and compression, revision surgery, the use of Kerrison rongeurs, bone spurs at decompression margins, increased abdominal pressure, and prolonged surgical duration; therefore, surgeons should enhance clinical awareness and vigilance during spine surgery, emphasizing the importance of meticulous intraoperative technique and comprehensive postoperative patient education to mitigate the incidence of this complication.

### 4.3 Treatment Approaches and Long‐Term Outcomes

While patients undergoing LPDT repair often experience initial postoperative satisfaction, the subsequent development of low‐pressure symptoms frequently necessitates bed rest, further hospitalization, and potentially additional surgery to address new, positional headaches distinct from their original lower back pain. This shift in symptomology often leads to a decline in overall clinical satisfaction [4, 17]. The management of LPDT involves both surgical and conservative approaches. Literature suggests that primary dural closure may not always be necessary. Successful outcomes have been achieved using techniques such as muscle patch grafts, microfibrillar collagen, the rotation of multifidus muscle pedicle flaps, fibrin sealants/fibrin glues, lumbar drains [4], dural sealants [25], blood patch [26]. When technically feasible, percutaneous CT‐guided fibrin glue injection or CT‐guided dural patching represent effective treatment options, particularly for small (< 5 mm) defects [27]. Endoscopic or transforaminal approaches can also be employed for dural tear repair [28].

Conservative treatment, including bed rest and wound care, can be effective in cases with mild symptoms and good wound healing. Bed rest minimizing abdominal pressure can facilitate closure of the defect; it's a key element in the treatment of persistent CSF leaks, as it can reduce the lumbar CSF pressure, thereby preventing CSF leakage [29]. Subsequently, the isolated pseudomeningocele may spontaneously resolve over time through reabsorption [30]. Various authors in the existing literature have reported different risk factors and management strategies for LPDT (Table 5). In this study, most patients underwent conservative treatment involving bed rest, which demonstrated significant clinical efficacy.

**TABLE 5:** Comparison of literature on risk factors associated with LPDT in lumbar surgery.

Author	Year	Journal	Sample size	Incidence	Risk factors of LPDT	Intervention
Khazim R [16]	2015	European Spine Journal	2052	0.83%	Spike of the bone left out at the edge	Surgical intervention in 88% (15/17) patients
Durand WM [19]	2018	Spine Journal	86,212	0.2%	Operative duration ≥ 250 min procedures with decompression only	Bed rest, epidural blood patch, reoperation
Kalidindi KKV [9]	2020	World Neurosurgery	1929	0.32%	Dura injured while arachnoid intact	Deep suturing, excision pseudomeningocele
Xu C [5]	2023	World Journal of Clinical Cases	Case reports		Continuous negative pressure suction, decreased dural ductility, malnutrition	Dural sealants
Halayqeh S [10]	2023	North American Spine Society Journal			Longer crew penetration	Epidural blood patch, fat graft, tisseel
Pando A [2]	2025	World Neurosurgery	11,636	No	Elderly patients, posterior approach, surgical extent	Suturing or patch placement intraoperation, conservation (20%)
Present study			2359	0.3%	Severe spinal canal stenosis, revision surgery, bone spurs, increased abdominal pressure	Wound pressure bandageand bed rest, dural repair

The long‐term outcomes of patients with LPDT vary based on the severity of the leakage and the treatment approach. In this study, 5 out of 7 patients with LPDT achieved good recovery, while 2 had poor outcomes. This underscores the importance of timely diagnosis and appropriate management to improve patient prognosis. To minimize the risk of LPDT, surgeons should employ meticulous surgical techniques and maintain a high index of suspicion for dural tears during lumbar surgery. Early recognition and intervention are crucial to prevent long‐term complications and ensure favorable outcomes [2, 31].

Although previous large‐sample studies have investigated CSF leakage after lumbar surgery, our research differs in a few key aspects. First, rather than treating all CSF leaks as a single group, we separated LPDT to compare them directly with early‐onset CSF leaks. This comparison showed that patients with LPDT generally experience worse clinical outcomes than those with early leaks—a finding rarely discussed in previous large cohorts. Second, we described the actual clinical progression from conservative to surgical treatment based on the timeline of symptoms, rather than relying solely on imaging findings. Finally, by analyzing these rare delayed cases within a cohort of 2359 patients, this study provides practical data to help guide the timing of interventions for late‐presenting CSF leaks.

### 4.4 Caution in Outcome Interpretation

Given the small sample size of LPDT cases (*n* = 7), our findings regarding treatment outcomes should be interpreted with caution and as preliminary observations rather than definitive conclusions about treatment efficacy.

## 5 Limitations and Strengths

This study is limited primarily by its single‐center, retrospective design and relatively small sample size, potentially impacting the generalizability and reliability of the findings. The absence of long‐term follow‐up data also restricts our ability to assess the long‐term effects of LPDT. The small number of LPDT cases (*n* = 7) limited our ability to perform robust statistical comparisons between LPDT and non‐LPDT CSF leak groups. The analyzes presented should be interpreted as descriptive rather than inferential for these subgroup comparisons. The inconsistency in applying MRI confirmation for all LPDT diagnoses, while reflecting real‐world clinical practice, represents a methodological limitation. Furthermore, the classification of some early postoperative CSF leaks (within 5 days) may include cases that could represent very early LPDT, potentially blurring the distinction between groups.

Future research could prioritize multi‐center collaboration, larger sample sizes, and extended follow‐up periods for LPDT patients to gain a more comprehensive understanding of its pathogenesis and treatment efficacy, ultimately strengthening the evidence base for clinical practice and advancing prevention and management strategies for postlumbar surgery CSF leaks.

## 6 Conclusion

CSF leakage due to late presentation of LPDT is a relatively rare but significant complication following lumbar surgery. This study found a 0.30% incidence of LPDT among lumbar surgery patients, with these cases demonstrating poorer outcomes compared to nondelayed CSF leaks. These findings highlight the importance of early recognition and appropriate management of LPDT to ensure favorable clinical outcomes. Surgeons should maintain a high index of suspicion for LPDT in patients presenting with relevant symptoms postoperatively.

## Author Contributions

X.Y., and D.T., and R.C. contributed to patient follow‐up, data collection, and statistical analysis. X.C., Y.Z., H.Z., Y.L., and L.D. participated in the surgeries and managed perioperative patient care. F.Z. and C.S. contributed to the study design and overall supervision of the project. All authors read and approved of the final manuscript.

## Funding

This study was supported by the National Key Research and Development Program of China (2023YFC3604400, 2023YFC3604404).

## Disclosure

The authors have nothing to report.

## Ethics Statement

The research protocol was approved by the Institutional Review Board of Peking University Third Hospital (IRB approval number: IRB00006761‐M2023296). Written informed consent was obtained from all patients.

## Consent

All authors have read and approved the final manuscript and consent to its publication.

## Conflicts of Interest

The authors declare no conflicts of interest.

## Data Availability

The datasets used during the current study are available from the corresponding author on reasonable request.
